# MicroRNAs involved in neoplastic transformation of liver cancer stem cells

**DOI:** 10.1186/1756-9966-29-169

**Published:** 2010-12-23

**Authors:** Ren Li, Niansong Qian, Kaishan Tao, Nan You, Xinchuan Wang, Kefeng Dou

**Affiliations:** 1 Hepato-Biliary Surgery Department, Xijing Hospital, the Forth Military Medical University, Western Changle Road, Xi'an, 710032, China; 2 Department of Hepatobiliary Surgery, Chinese People’s Liberation Army General Hospital, Fuxing Road, Peking, 100853, China

## Abstract

**Background:**

The existence of cancer stem cells in hepatocellular carcinoma (HCC) has been verified by characterizing side population (SP) cells based on efflux of Hoechst 33342 dye from stem cells. Recent advances in microRNA (miRNA) biology have revealed that miRNAs play an important role in embryonic development and tumorigenesis. However, it is still unclear which miRNAs participate in the neoplastic transformation of liver cancer stem cells (LCSCs) during hepatocarcinogenesis.

**Methods:**

To identify the unique set of miRNAs differentially regulated in LCSCs, we applied SP sorting to primary cultures of F344 rat HCC cancer cells treated with diethylnitrosamine (DEN) and normal syngenic fetal liver cells, and the stem-like characteristics of SP cells were verified through detecting expression of CD90.1, AFP and CK-7. Global miRNA expression profiles of two groups of SP cells were screened through microarray platform.

**Results:**

A total of 68 miRNAs, including miR-10b, miR-21, miR-470*, miR-34c-3p, and let-7i*, were identified as overexpressed in SP of HCC cells compared to fetal liver cells. Ten miRNAs were underexpressed, including miR-200a* and miR-148b*. These miRNAs were validated using stem-loop real-time reverse transcriptase polymerase chain reaction (RT-PCR).

**Conclusions:**

Our results suggest that LCSCs may have a distinct miRNA expression fingerprint during hepatocarcinogenesis. Dissecting these relationships will provide a new understanding of the function of miRNA in the process of neoplastic transformation of LCSCs.

## Background

Cancer stem cells (CSCs) have been identified in hematopoietic malignancies and in solid tumors, including hepatocellular carcinoma (HCC) [1,2]. The isolation and characterization of CSCs are usually based on the presence of known stem cell markers, i.e., CD133 in glioma [3] and CD44 and CD24 in breast cancer [4]. However, for many tissues, specific molecular markers of somatic stem cells are still unclear. Therefore, attempts have been made to identify CSCs in solid tumors through isolation of side population (SP) cells based on the efflux of Hoechst 33342 dye; such efflux is a specific property of stem cells [5]. The ability to isolate SP cells by cell sorting makes it possible to efficiently enrich both normal somatic stem cells and CSCs in vitro without the use of stem cell markers.

HCC is one of the most malignant tumors in existence. By using SP sorting, the existence of liver cancer stem cells in many established HCC cell lines has been verified [6-8]. However, few studies have focused on the isolation and characterization of SP cells isolated from primitive HCC cells. We conjectured that if normal hepatic stem cells (HSCs) and liver cancer stem cells (LCSCs) could be enriched through SP isolation, an in vitro model to determine whether HCC arises through the maturational arrest of HSCs could be developed.

MicroRNAs (miRNAs) are noncoding RNAs of 19 to 25 nucleotides in length that regulate gene expression by inducing translational inhibition and cleavage of their target mRNAs through base-pairing to partially or fully complementary sites [9]. Studies using the Dicer gene knockout mouse model have demonstrated that miRNAs may be critical regulators of the organogenesis of embryonic stem cells (ESC) [10,11]. Moreover, accumulated data suggest that dysregulation of miRNA occurs frequently in a variety of carcinomas, including those of the lung, colon, stomach, pancreas and liver [12]. The dual effects of miRNAs in both carcinogenesis and differentiation of normal stem cells strongly suggest that miRNA may be involved in the transformation of normal stem cells into cancer stem cells. Therefore, screening for differences in miRNA expression between normal HSCs and LCSCs should help to elucidate the complex molecular mechanism of hepatocarcinogenesis.

In this study, we applied SP analysis and sorting to F344 rat HCC cells induced with DEN and to syngenic rat day 14 embryonic fetal liver cells. After isolation of total RNA, microarray analysis of miRNA expression was performed in order to detect possible differences in expression levels of specific miRNAs in the two side populations. We found that 68 miRNAs were over-expressed in the side population of cancer cells compared to that obtained from fetal liver cells, while 10 miRNAs were relatively under-expressed. Partially dysregulated miRNAs were validated by real-time PCR analysis. Our results reveal that miRNAs may play an important function during the transformation of normal HSCs into LCSCs.

## Methods

### Animals and Chemical Carcinogenesis

Pregnant F344 rats and normal male F344 rats were purchased from the national rodent laboratory animal resources, Shanghai branch, China. All animals were housed in an air-conditioned room under specific pathogen-free (SPF) conditions at 22 ± 2°C and 55 ± 5% humidity with a 12 hour light/dark cycle. Food and tap water were available ad libitum. All operations were carried out under approval of Fourth Military Medical University Animal Ethics Committee. Primary HCCs were induced with DEN (80 mg/L in drinking water, Sigma, St. Louis, MO) for 6 weeks; animals were then provided with normal water until the appearance of typical tumor nodules in the liver, which usually occurred 10 to 12 weeks after treatment. After the rats were sacrificed under ether anesthesia, liver tissues were fixed with 4% paraformaldehyde, routinely processed and stained with hematoxylin and eosin (H&E) for histological examination by two pathologists, blinded to the results of the study, in order to verify the formation of HCC.

### Cell isolation and primary culture

Fetal liver cells were obtained from embryonic day 14 rat fetuses by the procedure of Nierhoff et al. [13]. The dissociated cells were inoculated onto culture plates with William's E medium (Sigma, St. Louis, MO) supplemented with 10% fetal calf serum (FCS) (Invitrogen), 100 U/mL penicillin G, 0.2 mg/mL streptomycin, and 500 ng/mL insulin. HCC cells were isolated from DEN-induced rat liver carcinomas. Briefly, tumor nodules in the liver were minced into pieces and digested by 0.5% collagenase type IV (Sigma, St. Louis, MO) at 37°C for 15 minutes. After filtration through 70 μm mesh, the dispersed cancer cells were collected by centrifugation and finally cultured in medium of the same composition as that used for fetal liver cells. The culture media were changed routinely every 3 days.

### Flow cytometry

To identify and isolate SP fractions, fetal liver cells and HCC cells were dissociated from culture plates with trypsin and EDTA, and pelleted by centrifugation. The cells were resuspended at 1 × 106/mL in pre-warmed HBSS with 2% bovine serum albumin (BSA) and 10 mmol/L HEPES. Hoechst 33342 dye was added to a final concentration of 5 mg/mL in the presence or absence of 50 μM verapamil (Sigma, USA), and cells were then incubated at 37°C for 90 minutes. After incubation, the cells were washed with ice-cold HBSS three times, and were further stained with FITC-conjugated anti-rat CD90.1 monoclonal antibody (Biolegend Co., USA). When staining was finished, propidium iodide (PI; final concentration 1 μg/ml) was added to identify viable cells. The cells were filtered through 80 μm mesh (Becton Dickinson Co., USA) to obtain a single cell suspension before analysis and sorting. Analysis and sorting were performed on a FACSVantage II (Becton Dickinson Co., USA). The Hoechst 33342 dye was excited at 355 nm and its fluorescence was dual-wavelength analyzed with emission for Hoechst blue at 445 nm, and Hoechst red at 650 nm.

### RNA isolation and miRNA microarray

Total RNA from two groups of SP cells was isolated using TRIZOL reagent (Invitrogen) according to the instructions of the supplier and was further purified using an RNeasy mini kit (Qiagen, Valencia, CA USA). The miRCURY Hy3/Hy5 labeling kit (Exiqon) was used to label purified miRNA with Hy3TM fluorescent dye. Labeled samples were hybridized on the miRCURY LNA (locked nucleic acid) Array (v.11.0, Exiqon, Denmark). Each sample was run in quadruplicate. Labeling efficiency was evaluated by analyzing signals from control spike-in capture probes. LNA-modified capture probes corresponding to human, mouse, and rat mature sense miRNA sequences based on Sanger's miRBASE version 13.0 were spotted onto the slides. The hybridization was carried out according to the manufacturer's instructions; a 635 nm laser was used to scan the slide using the Agilent G2505B. Data were analyzed using Genepix Pro 6.0.

### Statistical analysis

Signal intensities for each spot were calculated by subtracting local background (based on the median intensity of the area surrounding each spot) from total intensities. An average value of the three spot replicates of each miRNA was generated after data transformation (to convert any negative value to 0.01). Normalization was performed using a per-chip 50th percentile method that normalizes each chip on its median, allowing comparison among chips. In two class comparisons (embryonic hepatocytes SP vs. HCC SP), differentially expressed miRNAs were identified using the adjusted t-test procedure within the Significance Analysis of Microarrays (SAM). The SAM Excel plug-in used here calculated a score for each gene on the basis of the observed change in its expression relative to the standard deviation of all measurements. Because this was a multiple test, permutations were performed to calculate the false discovery rate (FDR) or q value. miRNAs with fold-changes greater than 2 or less than 0.5 were considered for further analysis. Hierarchical clustering was generated for both up-regulated and down-regulated genes and conditions using standard correlation as a measure of similarity.

### Real-time polymerase chain reaction (real-time RT-PCR) analysis

To compare the expression of AFP and CK-7 between SP and non-SP and validate the differential expression of miRNAs in SP fractions, we applied real-time RT-PCR analysis to sorted cells. Specially, stem-loop primers were used for reverse transcription reaction of miRNAs [14]. The complementary DNA (cDNA) underwent 40 rounds of amplification (Bio-Rad IQ5) as follows: 40 cycles of a 2-step PCR (95°C for 15 seconds, 60°C for 60 seconds) after initial denaturation (95°C for 10 minutes) with 2 μl of cDNA solution, 1× TaqMan SYBR Green Universal Mix PCR reaction buffer. The sequence of primers used for amplification is listed in Table 1. mRNA or miRNA levels were normalized using GAPDH or U6 RNA as a internal reference gene and compared with non-SP cells. The relative amount of each miRNA to U6 RNA was described using the 2^-∆∆Ct ^method [15].

**Table 1:** Reverse transcription and stem-loop primers for real-time RT-PCR

Gene name	Reverse transcription primer (5'-3')	PCR primers (5'-3') F: forward primer R: reverse primer
miR-21	GTCGTATCCAGTGCAGGGTCCGAGGTATTCGCACTGGATACGACTCAACA	F: CGCGCTAGCTTATCAGACTGA
		R: GTGCAGGGTCCGAGGT
miR-10b	GTCGTATCCAGTGCAGGGTCCGAGGTATTCGCACTGGATACGACCACAAA	F: CGTCGTACCCTGTAGAACCGA R: GTGCAGGGTCCGAGGT
miR-470*	GTCGTATCCAGTGCAGGGTCCGAGGTATTCGCACTGGATACGACTCTTCT	F: GTGCGAACCAGTACCTTTCTG R: GTGCAGGGTCCGAGGT
miR-34c-3p	GTCGTATCCAGTGCAGGGTCCGAGGTATTCGCACTGGATACGACCCTGGC	F:GGTGGAATCACTAACCACACG R: GTGCAGGGTCCGAGGT
let-7i*	GTCGTATCCAGTGCAGGGTCCGAGGTATTCGCACTGGATACGACAGCAAG	F: TAGTACTGCGCAAGCTACTGC R: GTGCAGGGTCCGAGGT
miR-200a*	GTCGTATCCAGTGCAGGGTCCGAGGTATTCGCACTGGATACGACTCCAGC	F: GAGTGCATCTTACCGGACAGT R: GTGCAGGGTCCGAGGT
miR-148b*	GTCGTATCCAGTGCAGGGTCCGAGGTATTCGCACTGGATACGACGCCTGA	F: GGCGCAAGTTCTGTTATACAC R: GTGCAGGGTCCGAGGT
U6	CGCTTCACGAATTTGCGTGTCAT	F: GCTTCGGCAGCACATATACTAAAAT R: CGCTTCACGAATTTGCGTGTCAT

### Western blotting analysis

Cells sorted by FACS were washed twice with ice-cold PBS and then incubated with ice-cold cell lysis buffer (1% Nonidet P-40, 50 mmol/L HEPES, pH7.4, 150 mmol/L NaCl, 2 mmol/L ethylenediaminetetraacetic acid, 2 mmol/L phenylmethylsulfonyl fluoride, 1 mmol/L sodium vanadate, 1 mmol/L sodium fluoride, and 1× protease inhibitor mixture) to extract protein. The protein concentrations of the lysates were measured using a Bradford protein assay kit (Bio-Rad). All samples were separated in 12% SDS polyacrylamide gels. Signal were revealed by primary antibodies and IRDye700-labeled secondary antibody. The signal intensity was determined by Odyssey Infrared Imaging System (LI-COR Bioscience, Lincoln, NE).

## Results

### SP cells are present in rat HCC cancer cell and fetal liver cells

**Figure 1:**
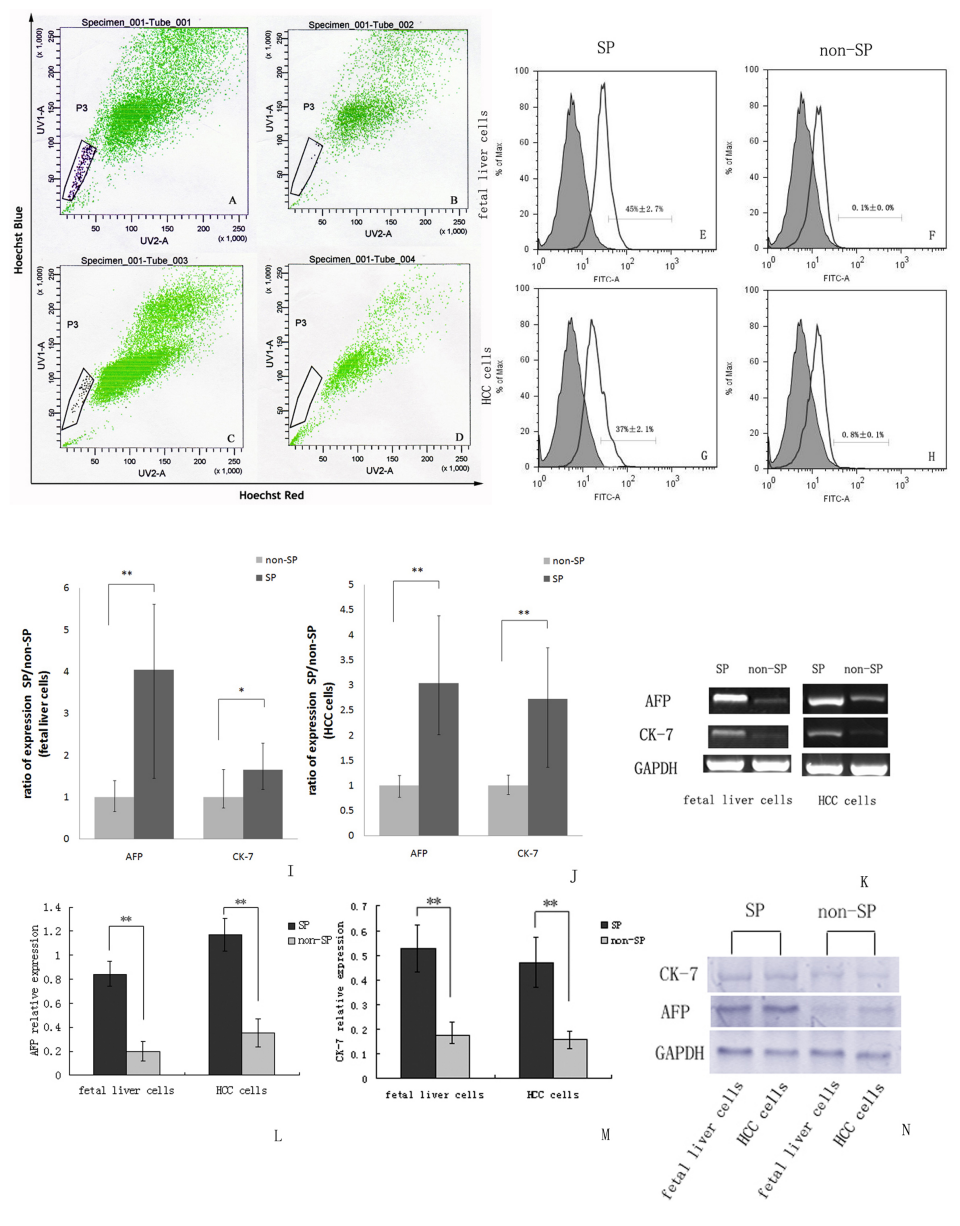
**SP cell and non-SP cells analysis**. (A and C) Representative side populations (SP) were identified in the P3 gate on the flow cytometry profile after the cells were stained with Hoechst 33342, (B and D): The SP cells in both HCC cells and fetal liver cells disappeared (0.0%) when cells are treated with 50 μM verapamil. (E-H) Analysis of stem cell marker expression on the surfaces of SP and non-SP cells. The number within each histogram represents the percentage of CD90.1 positive cells. (I-K) Quantitative analysis of AFP and CK-7 genes expression applied to sorted SP cells and non-SP cells by using Real-time RT-PCR. Data were normalized by using GAPDH housekeeping gene as endogenous control. (* P < 0.05, ** P < 0.01). (L-M) Western-blotting analysis of AFP and CK-7 protein expression in SP cells and non-SP cells. The relative expressions of protein were calculated through comparing with GAPDH protein.

The existence of the SP fraction in primary fetal liver cells and in HCC cells was confirmed by staining with Hoechst 33342 dye to generate a Hoechst blue-red profile. A small fraction of low-fluorescing cells in the lower-left region of each profile was gated as SP. The appearance of this fraction was blocked by verapamil, an inhibitor of transport via multidrug resistance proteins (Figure 1A-D). Both fetal liver cells and HCC cells contained a distinct fraction of SP cells. The SP of fetal liver cells was calculated to be 0.15% ± 0.02% (mean ± SEM), and that of HCC cells was calculated to be 0.20% ± 0.08%. Once identified, the cells in the SP gate were sorted into a centrifuge pipe by FACS.

### SP cells are enriched for markers of HSCs

To examine whether SP cells are enriched for characteristics of stem cells compared to the non-SP cells, we further characterized the SP cells from the fetal liver cells and HCC cells by analyzing the presence of markers known to be expressed commonly on the surface of HSCs. FACS analysis showed that CD90.1 positive cells made up 45% ± 2.7% of total SP from fetal liver cells, and 37% ± 2.1% of total SP from HCC cells. In contrast, only 0.1% ± 0.0% (fetal liver cells) and 0.8% ± 0.1% (HCC cells) were CD90.1 positive cells in non-SP fractions (Figure 1E-H). We next quantitatively compared the expression of AFP and CK-7 genes between sorted SP cells and non-SP cells. Real-time RT-PCR analysis revealed that AFP and CK-7 mRNA level in SP from the fetal liver cells were increased 4.3-fold and 1.9-fold, respectively compared to non-SP (Figure 1I). Similarly, in SP from the HCC cells, they were increased 3.6-fold and 2.7-fold, respectively (Figure 1J). Furthermore, the differentially gene expressing profile of AFP and CK-7 in sorted SP cells and non-SP cells also confirmed by using western-blotting analysis. As shown in Figure, the relative expression of AFP and CK-7 were 0.84 ± 0.10, 0.53 ± 0.01 in SP from the fetal liver cells. While they were only 0.20 ± 0.08 and 0.18 ± 0.05 in non-SP cells (Figure 1L). Similar results also could be seen in HCC cells group (SP: 1.17 ± 0.0.14, 0.47 ± 0.10; non-SP: 0.35 ± 0.12, 0.16 ± 0.04) (Figure 1M). These results indicate that the SP fraction appeared to be enriched with HSCs or LCSCs.

### miRNAs are differentially expressed in SP of fetal liver cells and HCC cells

**Figure 2:**
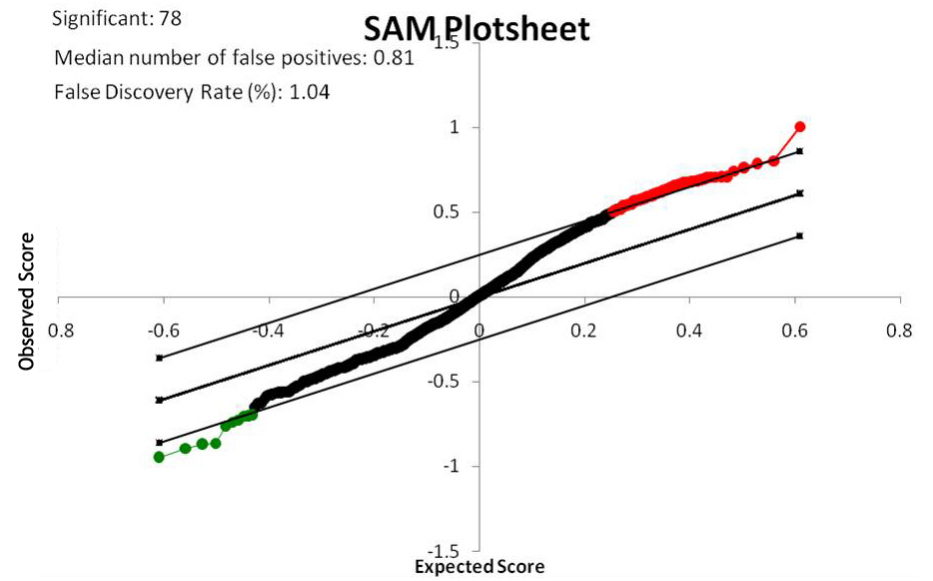
**SAM outputs**. SAM plotsheet outputs under the four sets of criteria: Δ = 0.25, fold change = 2. Conditions are indicated at the upper right corner of each plotsheet. The red, green, and black dots represent upregulated, downregulated, and insignificantly changed miRNAs, respectively. The upper and lower 45° degree lines indicate the Δ threshold boundaries. The number of significant miRNAs, median number of false positives, and false discovery rate (FDR) are indicated at the upper left corner of the plotsheet.

**Figure 3:**
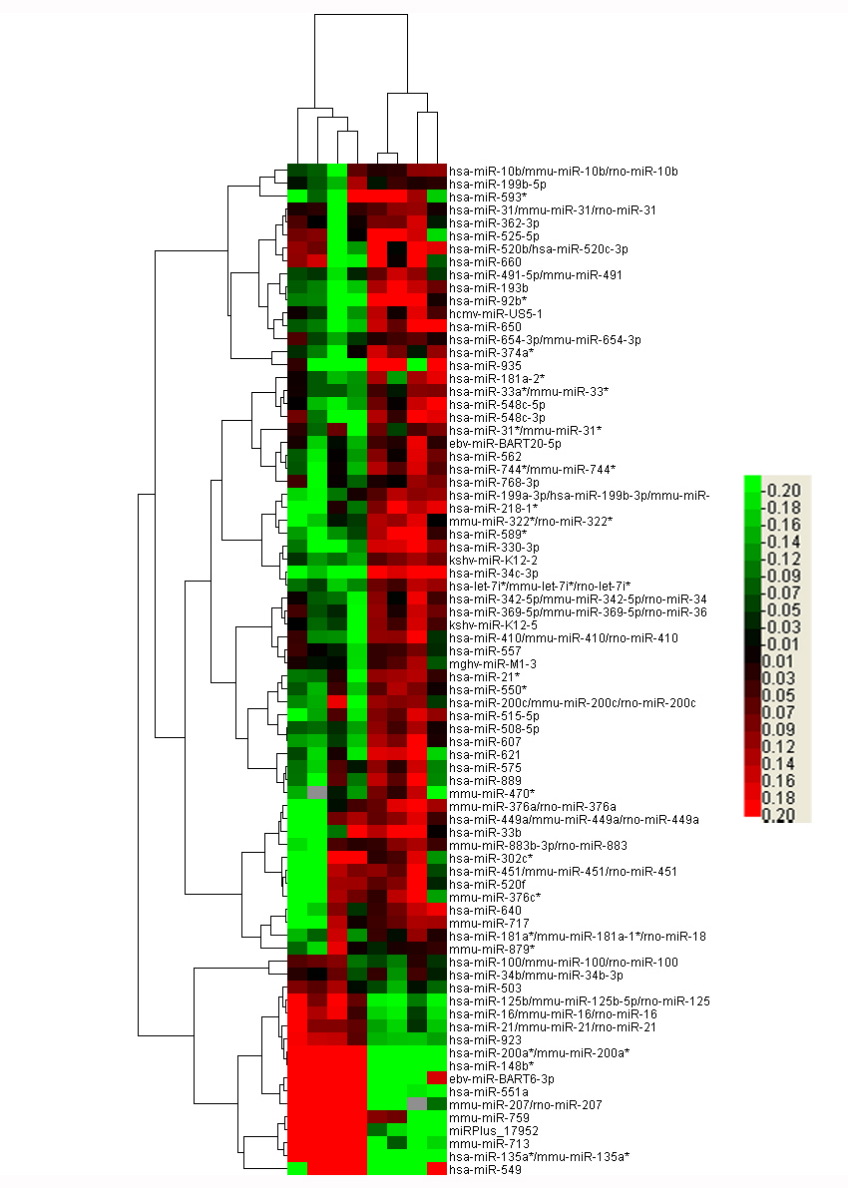
**Heat map of altered miRNA expression**. A heat map was generated using the expression ratios of 78 miRNAs that differed significantly in SP of HCC cells compared to fetal liver cells, according to significance analysis of microarrays (SAM). Red, overexpressed miRNAs; green, underexpressed miRNAs compared to counterparts. Relatedness in miRNA expression across samples is shown by a hierarchical tree on the Y axis through standard linkage.

To identify specific miRNAs that might function in neoplastic transformation of liver cancer stem cells, we analyzed global miRNA expression using miRCURY LNA Array that covered all microRNAs in miRBase. Slides were scanned using an Agilent G2565BA Microarray Scanner System and image analysis was carried out with ImaGene 7.0 software (BioDiscovery). The array data was further analyzed using SAM. Based on the fold-changes observed, 68 up-regulated miRNAs and 10 down-regulated miRNAs were identified in the SP of HCC cells compared to the fetal liver cells. A comprehensive list is shown in Table 2. The SAM analysis plot image is shown in Figure 2, and a hierarchical clustering image is shown in Figure 3.

**Table 2:** Partial list of miRNAs with significantly different levels detected in SP of HCC cells compared to fetal liver cells

microRNA	SAM score	Fold change	False discovery rate (FDR) %
hsa-miR-935	0.66	4.32	0.51
mmu-miR-10b	1.00	3.88	0.07
mmu-miR-21	0.80	2.96	0.00
mmu-miR-470*	0.69	2.81	0.00
hsa-miR-34c-3p	0.78	2.79	0.00
hsa-miR-650	0.76	2.71	0.00
hsa-miR-92b*	0.69	2.65	0.03
hsa-miR-193b	0.71	2.59	0.00
hsa-miR-374a*	0.68	2.58	0.24
hsa-miR-548c-3p	0.70	2.54	0.00
hsa-miR-33b	0.66	2.53	0.57
mmu-miR-199a-3p	0.71	2.52	0.00
hsa-miR-330-3p	0.71	2.51	0.00
mmu-miR-376a	0.69	2.48	0.13
mmu-miR-100	0.68	2.44	0.16
mmu-miR-717	0.66	2.36	0.62
mmu-miR-125b-5p	0.66	2.35	0.45
mmu-miR-449a	0.64	2.35	1.09
hsa-miR-21*	0.63	2.31	1.29
mmu-miR-883b-3p	0.63	2.29	1.20
mmu-miR-31	0.59	2.25	2.45
mmu-miR-34b-3p	0.57	2.14	3.43
mmu-let-7i*	0.55	2.02	4.66
hsa-miR-549	-0.70	0.05	2.84
mmu-miR-207	-0.86	0.23	6.02
mmu-miR-200a*	-0.94	0.29	1.22
mmu-miR-207	-0.86	0.23	0.60
hsa-miR-148b*	-0.76	0.36	2.72
mmu-miR-135a*	-0.69	0.38	2.92

### Validation of the differentially expressed miRNAs by qRT-PCR

**Figure 4:**
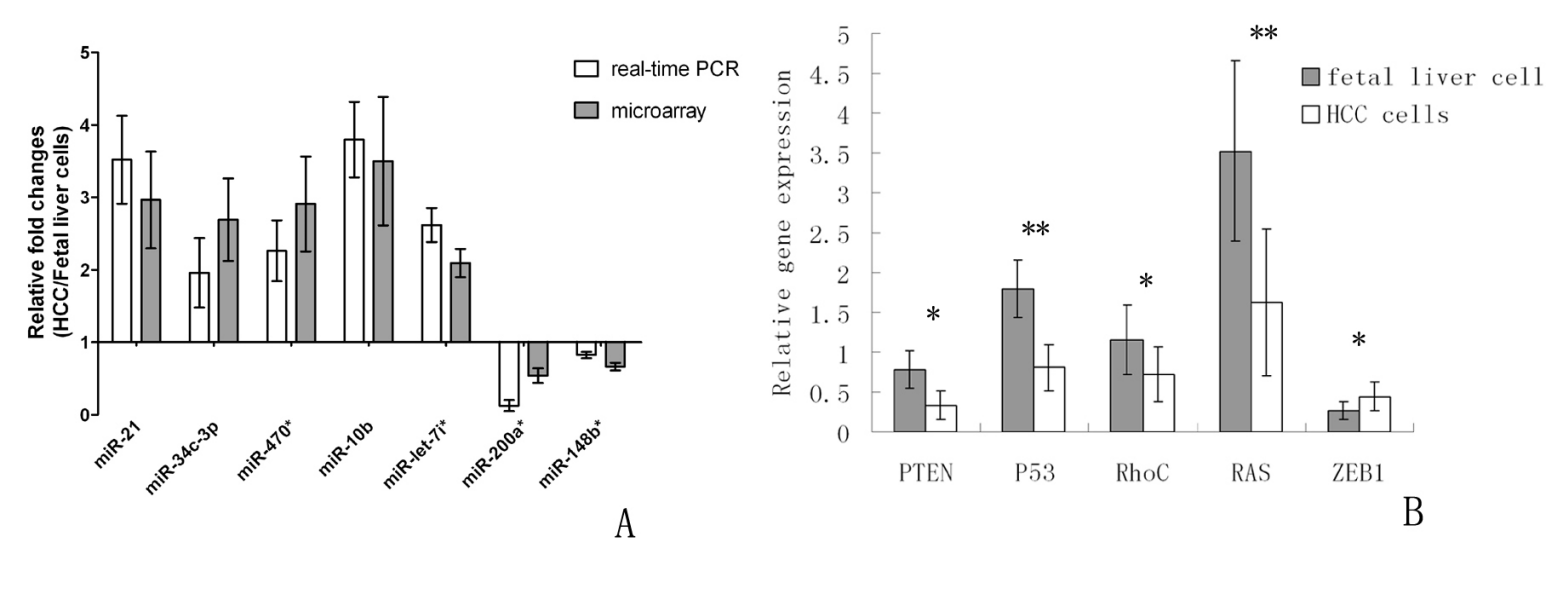
**Validation of microarray data using real-time RT-PCR**. (A) The levels of miR-21, miR-34c-3p, miR-470*, miR-10b and let-7i* are significantly increased, while the levels of miR-200a*, miR-148b are significantly decreased in the SP of HCC cells compared to the fetal liver cells, according to the results of microarray analysis (gray bar). Real-time RT-PCR analysis of these miRNAs using total RNA isolated from the SP fractions showed similar results (white bar). (B) Real-time analysis revealed that some known target genes of those partially validated miRNAs are also significantly differentially expressed between the SP sorted from the HCC cells and fetal liver cells (* P < 0.05; ** P < 0.01). The levels of target gene mRNA are inversely correlated with associated microRNA expression in SP cells.

Using a stringent cut-off of P < 0.05, we found significantly altered expression of only 7 of all rat miRNAs analyzed in SP of HCC cells. In detail, five miRNAs were significantly up-regulated (miR-21, miR-34c-3p, miR-470*, miR-10b, let-7i*) and two miRNAs significantly down-regulated in SP of HCC cells (miR-200a*, miR-148b*). miRNA-specific qRT-PCR was used to validate the significantly altered miRNAs from the miRNA microarray results. As shown in Figure 4A, the results showed that the expression levels of miR-21, miR-34c-3p, miR-16, miR-10b, and let-7i* in SP of HCC cells compared to SP of fetal liver cells were increased 3.5 ± 0.84, 2.1 ± 0.52, 2.2 ± 0.46, 3.9 ± 0.61, and 2.8 ± 0.25 -fold respectively, which were consistent with miRNA microarray results (P < 0.05). of the down-regulated miR-200a*, and miR-148b* in SP of HCC cells had the fold changes 0.1 ± 0.04, and 0.4 ± 0.08, respectively (P < 0.01).

To further confirm the differentially expressed miRNA, some known target genes expression of those validated miRNAs excluded miR-470* and miR-148b were detected in sorted SP cells and compared by using qRT-PCR between fetal liver cell and HCC cells. These target genes were PTEN (miR-21), P53 (miR-34c), Rho C (miR-10b), RAS (let-7i), and ZEB1 (miR-200a). As shown in Figure 4B, the relative gene expression of PTEN, P53, RhoC and RAS in SP from HCC cells were significantly lower than in fetal liver cells. On the contrary, the relative expression of ZEB1 gene in SP from HCC cells was higher than in fetal liver cells. Respectively, corresponding specific data were 0.78 ± 0.24 *vs *0.33 ± 0.18 (PTEN), 1.79 ± 0.36 *vs *0.81 ± 0.29 (P53), 1.16 ± 0.44 *vs *0.72 ± 0.34 (RhoC), 3.52 ± 1.13 *vs *1.62 ± 0.92 (RAS), and 0.27 ± 0.11 *vs *0.48 ± 0.13 (ZEB1). These data were indirectly validated the differentially expressing profile of those miRNAs in SP fractions between HCC cells and fetal liver cells.

## Discussion

There is a growing realization that many cancers may harbor a small population of cancer stem cells (CSCs). These cells not only exhibit stem cell characteristics, but also, importantly, are tumor-initiating cells and are responsible for cellular heterogeneity of cancer due to aberrant differentiation. According to the hierarchical model of cancer, the origin of the cancer stem cells may be long-lived somatic stem cells. Therefore, markers of "normal" stem cells are being sought with the expectation that these molecules are also expressed by cancer stem cells, and can be used to identify them. In fact, the specific markers of many somatic stem cells, e.g., HSCs, are still unidentified, and it is difficult to screen putative stem cell markers useful for isolation and characterization of liver cancer stem cells. Recently, however, a special common "marker" has been identified in the sense that characteristic stem-like cells possess an energy-dependent drug export property conferred by their high expression of ABC (ATP-binding cassette) membrane transporters. This property was first exploited by Goodell et al. [16] for isolation and analysis of hematopoietic stem cells based on their ability to efflux a fluorescent dye. Identified cells were termed a "side population". The SP fraction is a useful tool for cancer stem cell studies in solid tumors, especially when specific cell surface markers are unknown. In many gastrointestinal cancers and HCC cell lines, SP fraction cells have been identified and characterized by their capacity for self-renewal and their high tumorgenicity [17]. These studies demonstrated that SP can be used to enrich cancer stem cells in HCC. Moreover, it has been verified that normal HSCs (or 'oval cells') in rodents also express the side population phenotype defined by high expression of ABC transporter [18,19]. In the current study, we were able to identify a small SP component (0.10%-0.34%) in both fetal liver cells and HCC cancer cells of F344 rats. The percentage of SP cells we detected is similar to the percentages described in most previous reports of SP in human HCC cell lines[17]. To the best of our knowledge, this is the first report demonstrating the existence of SP cells in both fetal liver cells and in primary rodent HCC cancer cells induced by chemical carcinogens. Since the HCC cancer cells and fetal liver cells used in our study originated from the same inbred rat strain, the SP fractions enriched by screening both normal fetal liver and tumors for stem-like cell characteristics have high similarity in genetic background, thus providing a model for in vitro study of the mechanism of neoplastic transformation from normal HSCs into LCSCs. In contrast, it is difficult to accomplish this using SP cells sorted from many human HCC cell lines.

Increasing evidence has accumulated suggesting that many miRNAs play key roles in stem cell maintenance and differentiation. In ESC, disruption of the Dicer protein, an important enzyme in miRNA processing, leads to embryonic lethality [20]. Further evidence has also been provided by studies in some somatic stem cells showing that specific miRNA-based regulation is involved during organ and tissue development; e.g., a cardiac-enriched miRNA family was identified and demonstrated to have a critical role in the differentiation and proliferation of cardiac progenitor cells [21]. Additionally, experiments using isolated populations of hematopoietic stem cells have documented roles for specific miRNAs in HSC lineage differentiation, and evidence suggests that miRNAs are important for differentiation of somatic stem cells in several other tissues as well [22]. In addition to stem cell studies, microarray-based expression studies have also shown that aberrant expression of miRNAs occurs in several hematological and solid tumors including HCC [12]. In these malignancies, it has been shown that specific miRNAs can function either as oncogenes or as tumor suppressors during carcinogenesis [23]. Moreover, the aberrant miRNA expression profile correlated with particular tumor phenotypes can even be used to distinguish between normal tissue and tumors.

With the accumulation of evidence for "cancer stem cells", it is proposed that miRNAs might play a role in malignant transformation from normal stem cells into cancer stem cells. Recent studies have partially verified this hypothesis; e.g., let-7 miRNA expression can be observed in ESC and progenitor cells, but is absent in breast cancer stem cells. The reintroduction of let-7 into these cells causes differentiation and reduction of proliferation and tumor-forming ability. It has been demonstrated that in carcinogenesis, some miRNAs are likely to be instrumental in helping to control the delicate balance between the extraordinary ability of stem cells to self-renew, and their ability to differentiate for the purpose of development and tissue maintenance versus their potential for dysregulated growth and tumor formation [24]. In the present work, we have identified, for the first time, miRNA expression patterns that can unambiguously differentiate LCSCs and normal HSCs, though both were enriched in SP fractions and showed similar phenotypes. Our study demonstrates that the aberrant expression of some specific miRNAs may play a key regulatory role in the hepatocarcinogenesis of HSCs. Notably, the dysregulated miRNAs identified in our study are encoded in chromosomal regions that have frequent chromosomal instability during hepatocarcinogenesis, verified by previous comparative genomic hybridization. For example, the precursor sequences of the up-regulated miRNAs (miR-21, miR-10b) and down-regulated miR-148b* observed in our study are located at 17q23, 3q23 and 12q13. In these regions, chromosomal aberrations such as recurrent amplification, methylation or loss of heterozygosity have been detected in various clinicopathological HCC samples [25,26]. It has been shown that miRNA expression profiles of cancer stem cells are tissue-specific and tumor-specific. Moreover, comprehensive analysis of miRNA expression in diverse tumors has shown that miRNA genetic fingerprints can be used to accurately diagnose and predict tumor behavior [27,28]. While liver cancer stem cells are believed to be the tumor-initiating cells of HCC, we speculate that screening of circulating miRNAs in the serum could help to predict the presence of liver cancer stem cells and that such a procedure may be useful for early diagnosis of HCC.

Here we validated significant overexpression of miR-10b, miR-21, and miR-34c-3p in SP fractions of HCC compared to SP fractions of normal fetal liver cells. Notably, overexpression of these three miRNAs was previously shown to be an important factor in promoting cell invasion or proliferation in various tumor types. By performing real-time PCR, Sasayama et al. [29] found that miR-10b expression was upregulated in gliomas and that the expression of miR-10b was associated with higher-grade glioma. In glioma cells, miR-10b regulates the expression of mRNA for RhoC and urokinase-type plasminogen activator receptor (uPAR) via inhibition of translation of the mRNA encoding homeobox D 10 (HOXD 10), resulting in invasion and metastasis of glioma cells. Similarly, overexpression of miR-10b was also detected in metastatic breast cancer by Ma et al. [30], who showed that increased expression of miR-10b promoted cell migration and invasion. Additionally, it has been verified that miR-21 overexpression can down-regulate the Pdcd4 tumor suppressor and stimulate invasion, intravasation and metastasis in colorectal cancer [31]. Moreover, overexpression of miR-21 was also previously associated with poorly differentiated HCC, and this miRNA is known to participate in down-regulation of phosphatase and tensin homolog (PTEN) [32]. A different situation exists with other miRNAs such as miR-34c-3p, which is a member of the miR-34 family. Members of this family have been shown to be targets of the p53 gene, and to be involved in control of cell proliferation [33]. However, since inactivation of p53 is a critical event during hepatocarcinogenesis, it has been suggested that miRNAs play a central role in the aberrance of the p53 tumor suppressor network during neoplastic transformation of liver cancer stem cells, and that this is linked with multiple changes of phenotype such as cell cycle arrest and apoptosis.

A subset of miRNAs was also identified and shown to be significantly underexpressed in our study, including miR-200a and miR-148b*. Previous studies have linked the miR-200 family with the epithelial phenotype [34], and Korpal et al. [35] identified miR-200a as a suppressor of epithelial-mesenchymal transition (EMT) through direct targeting of ZEB1 and ZEB2 genes. EMT is a crucial process in the formation of various tissues and organs during embryonic development. Moreover, EMT is proposed to be a key step in the metastasis of epithelial-derived tumors including HCC. Thus, we hypothesize that the down-regulated miRNAs seen in this study may function as tumor suppressor genes during carcinogenesis. Although the exact target mRNA targets for many miRNAs are currently unknown, use of the TargetScan and MiRanda database to identify predicted target genes of the miRNAs shown to be up-regulated or down-regulated in our study could help to elucidate the neoplastic mechanism of liver cancer stem cells.

## Conclusions

This work provides an in vivo model for the study of mechanisms of neoplastic transformation of liver cancer stem cells by separately sorting SP fractions enriched with stem-like cells from primary rat HCC cancer cells and syngenic fetal liver cells. On the basis of this model, differences in miRNA expression profiles between LCSCs and normal HSCs were investigated using microarrays. This allowed us to identify miRNAs whose deregulation was closely correlated with the malignant phenotype of liver cancer stem cells, as distinguished from normal hepatic stem cells and from oncogene and tumor suppressor gene mutations. The gene and protein networks directly targeted and affected by these miRNAs that are likely to participate in tumorigenesis remain to be explored.

## Competing interests

The authors declare that they have no competing interests.

## Authors' contributions

LR and DKF designed the study. LR performed cell isolation and cultures. QNS performed the western-blotting and analyzed the data statistically. TKS performed quantitative PCR analysis for target genes of validated miRNAs. YN performed miRNAs microarray detection and data analysis. WXC accomplished quantitative PCR validation. LR wrote the main manuscript. DKF looked over the manuscript. All authors read and approved the final manuscript.
